# Influences of advanced glycosylation end products on the inner blood–retinal barrier in a co-culture cell model *in vitro*

**DOI:** 10.1515/biol-2020-0067

**Published:** 2020-08-24

**Authors:** Chen Yuan, Ya Mo, Jie Yang, Mei Zhang, Xuejun Xie

**Affiliations:** Eye School, Chengdu University of Traditional Chinese Medicine, Chengdu, Sichuan Province, People's Republic of China; Department of Ophthalmology, Hospital of Chengdu University of Traditional Chinese Medicine, Chengdu, 610072, Sichuan Province, People's Republic of China; Department of Neurology, Sichuan Academy of Medical Sciences & Sichuan Provincial People’s Hospital, Chengdu, Sichuan Province, People's Republic of China; School of Pharmacy, Chengdu University of Traditional Chinese Medicine & Key Laboratory of Standardization of Chinese Herbal Medicines of Ministry of Education & State Key Laboratory Breeding Base of Systematic Research, Development and Utilization of Chinese Medicine Resources, Chengdu, Sichuan Province, People's Republic of China

**Keywords:** advanced glycation end products, blood–retinal barrier, vascular endothelial growth factor, pigment epithelium-derived factor

## Abstract

Advanced glycosylation end products (AGEs) are harmful factors that can damage the inner blood–retinal barrier (iBRB). Rat retinal microvascular endothelial cells (RMECs) were isolated and cultured, and identified by anti-CD31 and von Willebrand factor polyclonal antibodies. Similarly, rat retinal Müller glial cells (RMGCs) were identified by H&E staining and with antibodies of glial fibrillary acidic protein and glutamine synthetase. The transepithelial electrical resistance (TEER) value was measured with a Millicell electrical resistance system to observe the leakage of the barrier. Transwell cell plates for co-culturing RMECs with RMGCs were used to construct an iBRB model, which was then tested with the addition of AGEs at final concentrations of 50 and 100 mg/L for 24, 48, and 72 h. AGEs in the *in vitro* iBRB model constructed by RMEC and RMGC co-culture led to the imbalance of the vascular endothelial growth factor (VEGF) and pigment epithelial derivative factor (PEDF), and the permeability of the RMEC layer increased because the TEER decreased in a dose- and time-dependent manner. AGEs increased VEGF but lowered PEDF in a dose- and time-dependent manner. The intervention with AGEs led to the change of the transendothelial resistance of the RMEC layer likely caused by the increased ratio of VEGF/PEDF.

## 1 Introduction

The blood–retinal barrier (BRB) is a special structure in the retina that regulates the exchange of substances inside and outside the blood vessels of the retina. Humans and certain species of animals have dual blood supply systems: retinal blood vessels and choroidal blood vessels. The BRBs corresponding to the two vascular systems are the inner blood–retinal barrier (iBRB) and the outer blood–retinal barrier. The iBRB is composed of retinal capillary endothelial cells, pericytes, which are tightly associated with the adjacent basement membrane surrounded by astrocytes, and Müller cells as a structural scaffolding for the iBRB [1]. This barrier prevents the free diffusion between the circulating blood and the neural retina, but it provides nutrition to the retina and removes endogenous organisms and foreign objects from the retina, such as inflammatory lymphocytes, blood-borne pathogens, excessive enzymes, and other toxic compounds. Due to the presence of the BRB, the drugs entering the retina are limited, and such a barrier has evolved well enough to provide protection for the retinal microenvironment, and it restricts 98% of clinically validated low molecular weight drugs [2]. Therefore, using the iBRB model would help us to understand more the retinal function. As a matter of fact, *in vivo* experiments can maintain the complete structure of the iBRB, and the information obtained is closer to reality, but the factors affecting the experiment are not easy to control, and the operation is tedious, time-consuming, and economically expensive, and hence is not suitable for large-scale drug screening [3]. In recent years, more attention has been paid to the development of convenient, fast, economical, and more corresponding *in vitro* models [4]. To accurately predict how a drug will behave *in vivo* through an *in vitro* model, one must have as many iBRB features as possible. With an ideal *in vitro* model, the results or conclusions may be closer to the situation *in vivo*, and the predictions may have more practical significance [5].

Retina is a complex structure composed of many types of cells, including retinal microvascular endothelial cells (RMECs), retinal pigment epithelial cells, neurons, retinal Müller glial cells (RMGCs), pericytes, and other types of cells, and an *in vitro* model should simulate the retinal structure and function; thus, the co-culture of different types of retinal cells with RMECs has advantages over culturing RMECs alone. The glial cells in the vertebrate retina, mainly made of RMGCs, play important roles in maintaining the normal structure, metabolism, and function of the retina. These glial cells contact the vitreous cavity through the enlarged basal and the subretinal space by the microvilli and act as a skeletal support in the formation of the BRB [6], and a variety of cytokines regulate the permeability of the retinal barrier [7]. However, most of the *in vitro* studies of the iBRB have been based on RMEC culture models. RMECs are the sites of the early onset of diabetic retinopathy (DR), and the damage of RMECs can lead to the collapse of the iBRB, leading to the progress of DR. Therefore, RMEC culture is often used as a model for studying human pathogenesis of DR and evaluating the effects of drugs [8].

Advanced glycosylation end products (AGEs) from chronic hyperglycemia are involved in the occurrence and development of chronic complications of diabetes. Once AGEs are formed, they are not easily degraded. Under the conditions of hyperglycemia, AGEs cause endothelial dysfunction by inducing abnormal cross-linking of extracellular matrix proteins and interacting with their receptors, triggering intracellular signaling cascades [9,10,11]. Hyperglycemia induces oxidative stress, hypoxia, white blood cell arrest, vasoconstriction, inflammation, and angiogenesis in the retina, further leading to vascular stiffness and dysfunction, pericyte apoptosis, and intraretinal destruction [12]. The destruction of the iBRB is the early and main pathophysiological basis of retinal vascular disease caused by AGEs. The mechanism of iBRB injury is closely related to vascular endothelial growth factor (VEGF) and pigment epithelial derivative factor (PEDF). The effects of AGEs on iBRB models of rat RMEC plus RMGC co-culture *in vitro* need to be investigated through the transepithelial electrical resistance (TEER), which is a good indicator of the integrity of the endothelial tissue barrier and provides a certain experimental basis for *in vivo* research on human DR and other retinal vascular diseases [13].

## 2 Materials and methods

### 2.1 Rat RMEC isolation, culture, and identification

A single newborn 7-day-old Sprague Dawley (SD) rat (from the Laboratory Animal Center, Third Military Medical University, Chongqing, China) was sacrificed by cervical dislocation. The eyeballs were subsequently removed under aseptic conditions, soaked in 75% ethanol for 15 min, rinsed with phosphate-buffered saline containing penicillin/streptomycin three times, and placed in an appropriate serum-free DMEM solution containing penicillin/streptomycin (Gibco, USA), and the retinas were collected under a stereo microscope. The retinas were cut into small pieces in a Petri dish, digested with 0.1% type I collagenase at 37°C for 30 min, and then filtered through a 200-mesh stainless steel cell sieve. The filtrates were centrifuged at 1,000 rpm for 5 min and resuspended in DMEM (Gibco, USA) containing 10% fetal bovine serum (FBS). CD31 antibody-coated immunomagnetic beads (eBioscience, USA) were added and incubated at 4°C for 30 min. After centrifugation at 1,000 rpm for 5 min, the cells were resuspended in 4 mL of DMEM and transferred to a test tube, which was then placed in a magnet for 2 min to collect the cells. The cells were seeded into poly-l-lysine-coated plates and cultured at 37°C in a 5% CO_2_ incubator (Sanyo, Japan) after washing the magnetic bead-bound cells four times with 10% FBS DMEM. The culture medium was changed every other day, and the cells were passaged with 0.25% trypsin (Gibco, USA) and 0.02% EDTA (1:1) digestion solution when cell fusion reached 70–80%. The RMECs out of rat retinal vascular endothelial cells (RRVECs) were recovered after identification by CD31/PE antibody and mouse IgG (eBioscience, USA) flow cytometry and by using the Von Willebrand factor (VWF) antibody (dilution ratio 1:400, Abcam, USA) with HRP-labeled secondary antibody at a dilution of 1:50 (Beyotime, China). The immunostained cells were observed with 3,3'-Diaminobenzidine (DAB) and hematoxylin staining under a microscope (Olympus, Japan).


**Ethical approval:** The research related to animal use has been complied with all the relevant national regulations and institutional policies for the care and use of animals and has been approved by the Ethics Committee of Chengdu University of Traditional Chinese Medicine.

### 2.2 Rat RMGC isolation, culture, and identification

A similar procedure was used until the retina was gently collected under a stereo microscope. The retina was blown repeatedly to form a chyle-like mixture, which was subsequently inoculated into a gelatin-coated culture flask, and the culture medium was changed after 72 h. After the 0.25% trypsin digestion was terminated with DMEM containing FBS, the cells were collected by centrifugation at 1,000 rpm for 10 min and gently pipetted to make the mixture even, and a drop of the cell suspension was taken to count the cells to adjust the amount of culture medium appropriately to a cell density of 1 × 10^6^/mL. The cells were aliquoted and routinely cultured in a 3 mL flask in a 5% CO_2_, 37°C incubator. When the cell fusion reached 80%, the cells were fixed with 4% paraformaldehyde and subjected to hematoxylin and eosin (H&E) staining, and RMGCs were screened and identified by glial fibrillary acidic protein (GFAP) or glutamine synthetase (GS) antibody (Abcam, USA) immunocytochemical staining.

### 2.3 Construction of the *in vitro* iBRB model

RMECs and RMGCs were used to simulate iBRB models *in vitro* after the characterization. The third generation cells of primary rat RMECs were collected at 70% fusion and cultured in a serum-free medium for 24 h. The second passage cells of rat RMGCs were collected at close to 70% fusion and cultured in a normal RMEC medium (DMEM containing 20% FBS and 100 U/mL penicillin + 100 U/mL streptomycin) for 24 h. In the meantime, a microporous membrane was pre-coated with 1% gelatin in a Transwell chamber (Corning, USA), and a single-cell suspension of RMGCs was seeded at a density of 1 × 10^4^/cm^2^ at the bottom of the Transwell chamber (lower compartment) and incubated for 2 h at 37°C. The Transwell chamber was turned over in a six-well plate after most of the RMGCs attached to the wall. Then, RMECs were seeded at the bottom of the Transwell chamber as the upper compartment at a density of 2 × 10^4^/cm^2^. The model was evaluated by recording the TEER of the RMEC layer over 3–13 days (Figure 3a).

### 2.4 TEER measurement of the RMEC layer in the iBRB model

The TEER was measured on the RMEC layer of the iBRB cell co-culture *in vitro* using a MilliCell® ERS-2 voltohmmeter. The measurement of each well’s resistance took place at three different points selected at random. The average value *R*_*t*_ was recorded, and the background resistance formed in the cell-free culture pool as the blank value *R*_0_ was used to calculate the resistance value of the entire endothelial cell, using the formula TEER = (*R*_*t*_ − *R*_0_) × *S*, where *S* is the effective surface area of the film. Transwells with TEER values greater than 90 Ω cm^2^ were selected for further use. The TEER value was measured every other day. A decreased value of TEER indicates that the permeability is increased.

### 2.5 Enzyme-linked immunosorbent assay (ELISA)

An ELISA was used to determine the levels of VEGF and PEDF in the presence of AGEs (Bioss Co., Beijing, China) at final concentrations of 50 and 100 mg/L for 24, 48, and 72 h. After AGE treatment in each experimental group, the cell supernatant was collected following centrifugation at 3,000 rpm for 20 min and used for the ELISA of VEGF and PEDF (BioTek, USA) according to the manufacturer’s instructions. The absorbance was measured at a wavelength of 450 nm, and the concentrations of VEGF and PEDF were calculated according to the linear regression equation of the standard curve and the dilution factors.

### 2.6 Statistical analysis

SPSS 21.0 (IBM Corp., Armonk, NY, USA) was used for statistical analysis. All data were expressed as mean ± standard deviation. Comparisons between groups were analyzed using one-way analysis of variance and *post hoc* tests. Univariate analysis of variance was used for comparison of the same index among multiple groups; the least significant difference test was used for pairwise comparison between multiple groups. All experiments for statistical analysis were repeated six times. *P* < 0.05 indicated that the difference was statistically significant.

## 3 Results

### 3.1 Isolation and identification of rat RMECs

**Figure 1:**
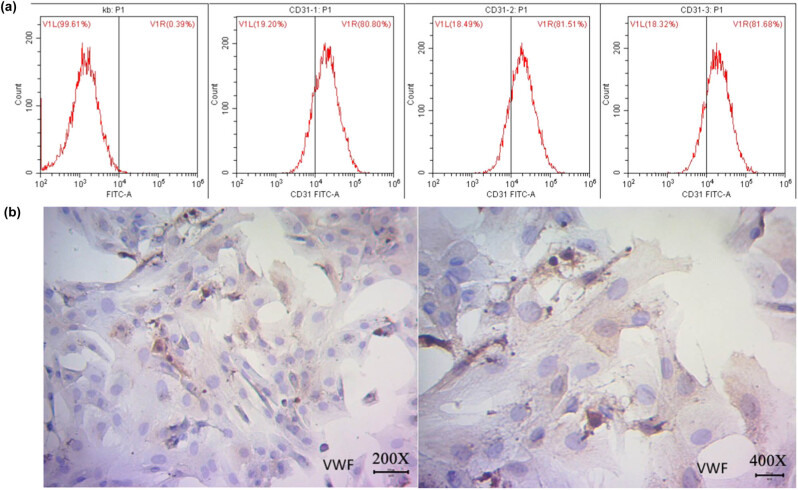
Isolation and identification of RMECs from RRVECs. RRVECs were isolated and cultured from the retina of a 7-day-old neonatal SD rat, and RMECs were verified after three passages and flow cytometry. (a) Verification of RMECs using flow cytometry with CD31 antibody. Kb - the blank control without antibody; P1 - the cell content after removal of cell debris; V1L - the content of the negative cells which were not labeled by CD31; V1R - the content of the CD31 positive cells, RMECs > 80% among RRVECs. (b) Immunological verification of RMECs with a polyclonal antibody against the VWF.

RMECs were isolated and cultured from a newborn SD rat. As measured by CD31 flow cytometry, the purity of RMECs reached 81% (Figure 1a). The cells were also identified using VWF polyclonal antibody immunohistochemistry (Figure 1b). These results showed that RMECs were successfully isolated and cultured because CD31 is a unique biomarker for RMECs. Similar to CD31, VWF is currently a commonly used vascular endothelial cell marker. However, VWF is only abundantly expressed in vascular endothelial cells, and this protein is not present in smooth muscle cells and fibroblasts, which ensured that the isolated RMECs have the characteristics of pericytes [14].

### 3.2 Isolation and identification of rat RMGCs

**Figure 2:**
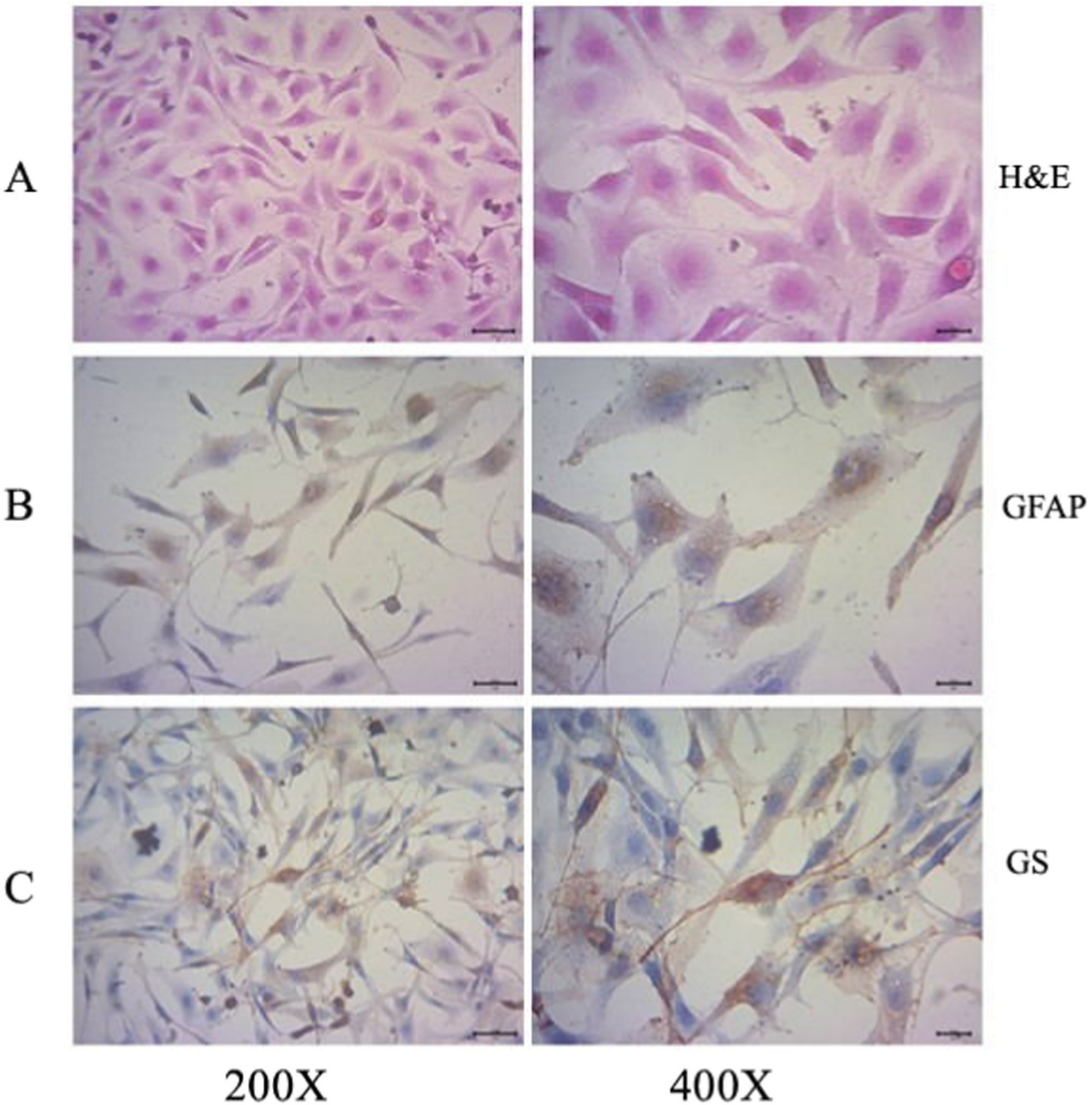
Isolation and identification of RMGCs from rat retinal Müller cells (RRMCs). RRMCs were isolated and cultured from the retina of a 7-day-old newborn SD rat, and RMGCs were verified using (a) H&E staining, (b) GFAP, and (c) GS immunocytochemical staining after two passages. RMGCs among RRMCs were indicated with GFAP and GS positive staining.

RMGCs were isolated and cultured from a newborn SD rat. H&E staining showed that the cell shape was narrow and long, the cytoplasm was abundant, the membrane was clear, and a deep red, large, and elliptical nucleus was located in the center of the cell in a mononucleated or multinucleated form (Figure 2a). More than 85% of the glial cells were GFAP positive, and brown-yellow silk-like structures were visible in the cytoplasm (Figure 2b), and more than 80% of the cells were positive for GS staining, especially the nucleus and the surrounding cytosol after 5 days of culture (Figure 2c). The results showed that RMGCs were successfully characterized and cultured because GFAP and GS are biomarkers for RMGCs.

### 3.3 Changes of TEER in the iBRB model after treatment with AGEs

**Figure 3:**
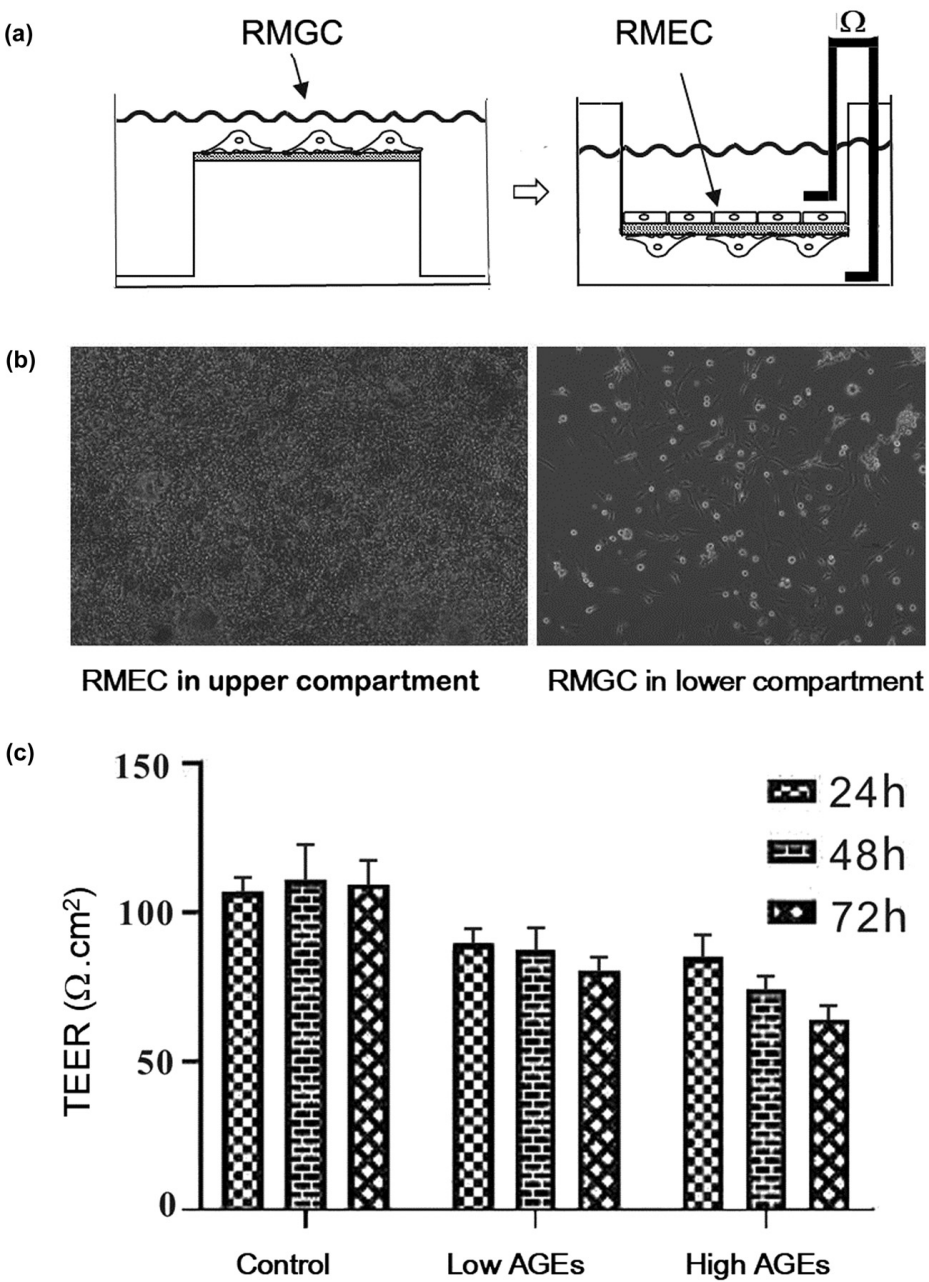
TEER changes in the iBRB model caused by AGEs. (a) Diagram illustrating the iBRB model *in vitro*. (b) Transwell assay; RMEC and RMGC layers (100×) of *in vitro* co-culture. (c) TEER measurement of the iBRB model *in vitro* conducted after treatment with indicated AGEs for 24, 48, and 72 h.

The cell layers in the *in vitro* iBRB model made of RMECs were established in the upper compartment (Figure 3b) because RMECs form the peripheral boundary membrane through interacting with RMGCs, where RMGCs provide nutrients and structural support and maintain the extracellular environment. The co-culture model of RMECs and RMGCs showed that the TEER of the RMEC layer was stable until 13 days, indicating that the model was successfully established, which was then treated with AGEs at final concentrations of 50 and 100 mg/L for 24, 48, and 72 h, and the TEER values of the RMEC layer were measured. Compared with the normal control group, the permeability of the RMEC layer of the model was increased after treatment with AGEs. The intervention with AGEs led to a decrease of TEER across the RMEC layer in a dose- and time-dependent manner (Figure 3c).

### 3.4 Changes of VEGF induced by AGEs in iBRB models *in vitro*

**Figure 4:**
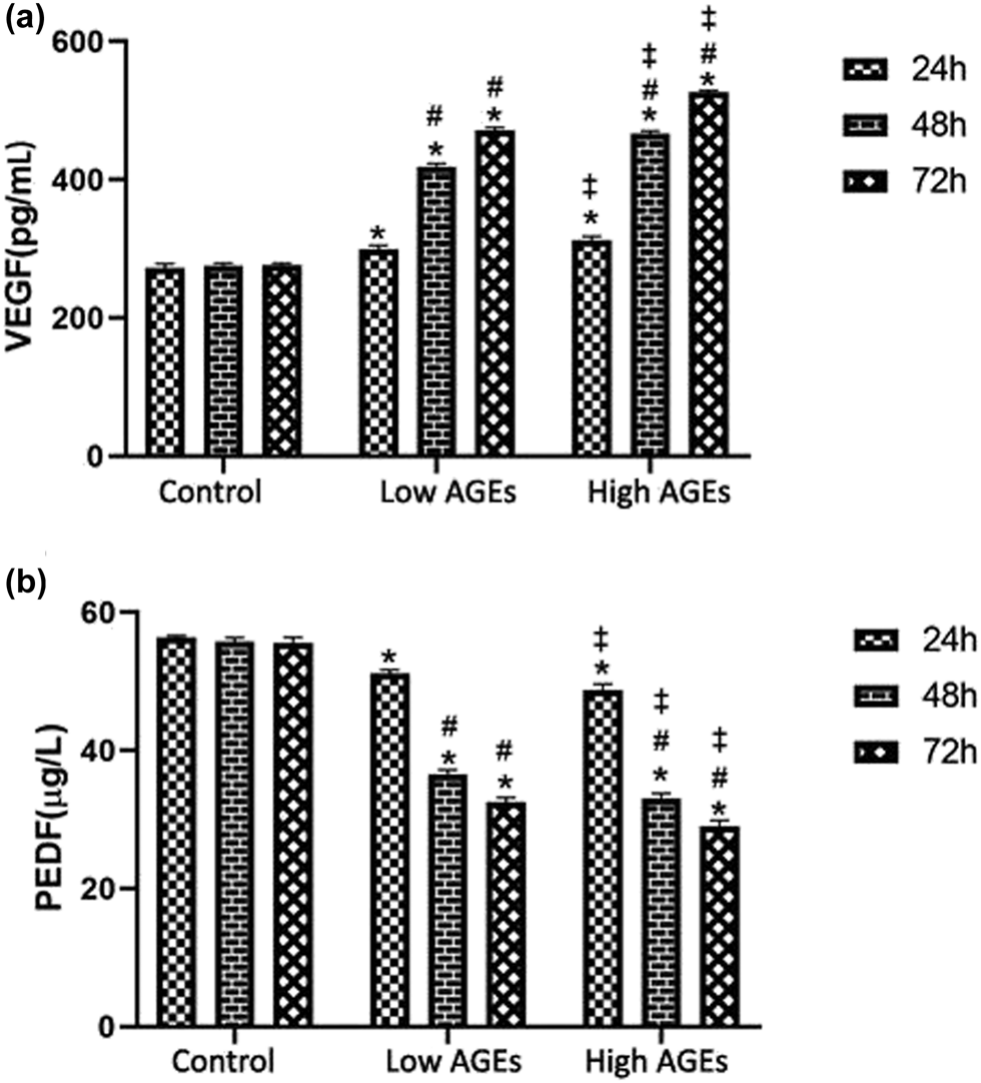
Changes of VEGF and PEDF in the iBRB-Müller cell co-culture due to AGEs. (a) VEGF in the media of the *in vitro* iBRB-Müller cell co-culture model determined using ELISA after treatment with indicated AGEs for 24, 48, and 72 h. (b) PEDF in the media of the *in vitro* iBRB-Müller cell co-culture model determined using ELISA after treatment with indicated AGEs for 24, 48, and 72 h. *P < 0.05 compared with the vehicle control group; #*P* < 0.05 compared with the low-dose group at 24 h earlier; ‡*P* < 0.05 compared with the high-dose group, at the same time point.

VEGF was measured by ELISA after treating the RMEC–RMGC cell co-culture with AGEs at final concentrations of 50 and 100 mg/L for 24, 48, and 72 h. The difference in VEGF concentrations between the different experimental groups in the same period was significant (*P* < 0.05). In the low-AGE group and the high-AGE group, the VEGF levels at different periods were compared, and *P* < 0.05. The results showed that treatment with AGEs resulted in a significant increase in VEGF levels in the RMEC–RMGC co-culture model in a dose- and time-dependent manner (Figure 4a).

### 3.5 Changes of PEDF induced by AGEs in the iBRB model *in vitro*

Similarly, ELISA was used to determine the level of PEDF in the RMEC–RMGC cell co-culture models treated with AGEs at final concentrations of 50 and 100 mg/L for 24, 48 and 72 h. The results showed that the difference in PEDF concentrations between control and experimental groups at any time period was statistically significant, *P* < 0.05. The results showed that compared with the normal control group, treatment with AGEs resulted in a significant reduction in PEDF levels in the co-culture model in a dose- and time-dependent manner (Figure 4b).

### 3.6 Changes of the ratio of VEGF/PEDF *in vitro* induced by AGEs

**Figure 5:**
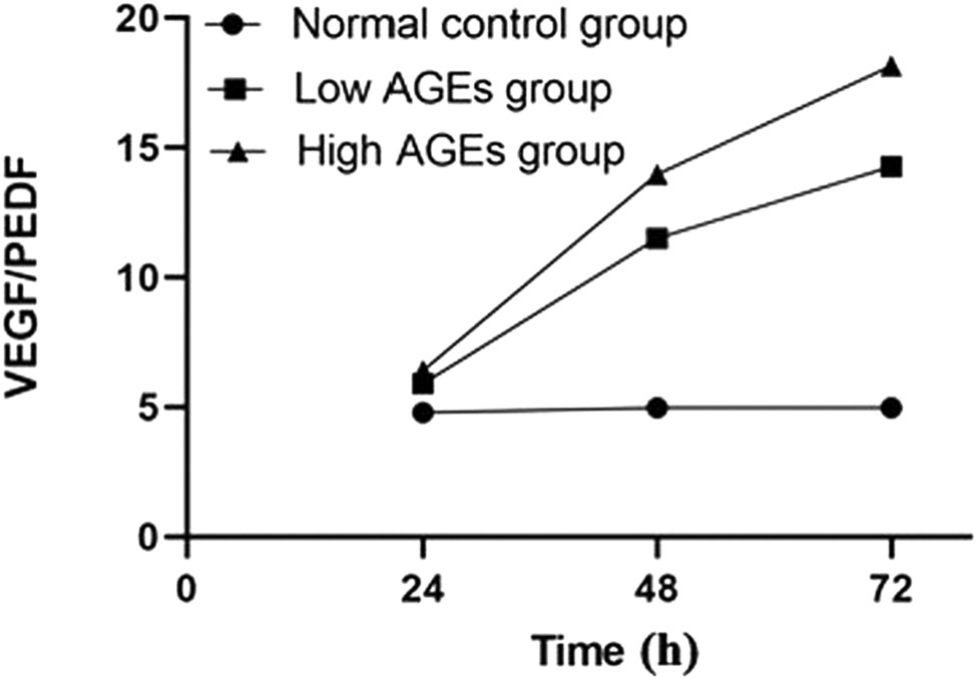
VEGF/PEDF in the RMEC layer of the *in vitro* iBRB-Müller cell co-culture after AGE treatment. VEGF and PEDF in the media of the *in vitro* iBRB-Müller cell co-culture were determined simultaneously using ELISA after treatment with indicated AGEs for 24, 48, and 72 h, and the ratios of the average values of the two cytokines were plotted against time points.

VEGF and PEDF protein concentrations were simultaneously measured with ELISA after treating RMEC–RMGC co-cultivation models with AGEs at final concentrations of 50 and 100 mg/L for 24, 48, and 72 h. The ratios of the normal control group at 24, 48, and 72 h were almost the same, and the amounts of VEGF and PEDF were in a relatively balanced state. However, for both the low-AGE group and the high-AGE group, they were higher than those in the normal control group. The ratio for the low-AGE group and the high-AGE group increased at 48 h and continued to increase at 72 h. The results showed that compared with the normal control group, the treatment of AGEs caused a proportional increase of VEGF in the co-culture, which means it led to the inhibition of PEDF in a time-dependent manner (Figure 5). It is worthy to mention that the measurement of VEGF and PEDF had been carried out in one experiment for measuring VEGF, PEDF, and VEGF/PEDF at the same time, and also in separate experiments, which all were included in statistical analysis.

## 4 Discussion

It has been reported that Müller cells induce a barrier in vascular endothelial cells [15], and Müller cells play an important role in the formation of the retinal vascular barrier. Therefore, RMGCs play an active role in the induction and maintenance of RMEC transition from non-barrier cells to barrier cells [16,17]. Studies have shown that the most important pathological basis and morphological changes in early DR start in the iBRB, and the structure of the iBRB will be damaged if it is directly exposed to hyperglycemic conditions [18]. A large number of studies have shown that AGEs are widely present in various eyeball tissues such as the cornea, retina, vitreous body, lens, Bruch’s membrane, sclera, and optic nerve, directly or indirectly leading to a series of eye diseases including retinopathy [19]. AGEs not only cause platelet activation and aggregation but also stimulate prothrombin activity by increasing the expression of tissue factors, which lead to thrombosis [20]. In the meantime, AGEs have been shown to inhibit the production of prostacyclin and induce the production of plasminogen activator inhibitor-1 in endothelial cells through interaction with the receptor of AGEs [21]. Therefore, AGEs may cause platelet aggregation and fibrin stabilization leading to thrombosis, thereby promoting vascular damage in diabetes. We demonstrated that co-cultured RMECs and RMGCs were stabilized because the TEER remained higher than 90 Ω cm^2^ after the 11th day, which indicated that the barrier was successfully established. With the established iBRB model, the addition of AGEs within the effective concentration range of 50–100 mg/L led to iBRB damage [22], and this was indicated by the reduced TEER value.

Due to the large accumulation of AGEs, the iBRB can be easily destroyed, and DR aggravates resulting in vascular edema and nerve tissue damage, eventually leading to vision loss [23]. Overexpression of VEGF under pathological conditions causes increased vascular permeability and neovascularization. At present, a large number of clinical and animal experiments have shown that high amounts of glucose in blood and tissues stimulate the large-scale production of VEGF, which is one of the mechanisms of DR development, and high levels of VEGF cause retinal iBRB damage and subsequently retinal exudation, hemorrhage, edema, neovascularization, and early DR. VEGF and PEDF are a group of major cytokines that are most closely related to the occurrence and development of DR. Therefore, the cytokines VEGF and PEDF were tested separately and combinedly analyzed. In a normal eye, Müller cells, pericytes, pigment epithelial cells, and endothelial cells of the retina secrete VEGF, but the expression levels are low. The low secretion is conducive to the maintenance of normal blood vessel function. In this experiment, the VEGF concentration in experimental groups in the same period was increased from the low-AGE group to the high-AGE group at different periods (*P* < 0.05), and our observation is consistent with previous related studies: the higher the AGE concentration, the higher the VEGF in the tested range [24].

PEDF is a polypeptide mainly from retinal pigment epithelium and retinal Müller cells, and it inhibits vascular leakage and vascular regeneration. A large number of studies have shown that the expression of PEDF in the eyes of patients with DR is significantly reduced, and this situation is more pronounced in patients with proliferative DR. Both cell culture and animal experiments have confirmed that AGEs can significantly improve their functions after adding exogenous PEDF. However, the accumulation of AGEs in diabetes mellitus significantly inhibits the expression of PEDF mRNA, and the transcription and expression of PEDF are reduced [25]. The oxidative stress aggravates injury in a high-glucose environment, promotes damage to the iBRB structure, and thereby accelerates retinal edema and the formation of new blood vessels [26]. In this experiment, the PEDF concentrations between control and experimental groups in the same period were significantly different, and the difference in PEDF concentrations in low-AGE and high-AGE groups at different periods was also significant. This shows that higher AGE concentration and a longer time lowered the concentration of PEDF.

The mechanism of iBRB injury under diabetic conditions is related to VEGF and PEDF. VEGF promotes early iBRB injury in DR and increases the leakage of retinopathy vessels; on the other hand, PEDF has the function of inhibiting vascular leakage of DR with a protective effect on the iBRB. Under physiological conditions, there is a balanced relationship between PEDF and VEGF. PEDF inhibits the increase of VEGF’s vascular permeability and angiogenic potential [26]. In this experiment, we showed that the ratios of the normal control group at different time points were almost the same, indicating that the levels of VEGF and PEDF proteins were in a relatively balanced state. But the ratios of VEGF/PEDF changed in the low-AGE group and the high-AGE group at different time points, both increased at 48 h and continued to increase at 72 h. This showed that the balance of VEGF and PEDF was lost after the addition of AGEs, and the expression ratio *in vitro* revealed a similar expression pattern to that *in vivo*. Our results also support the early finding that PEDF inhibits the VEGF-mediated angiogenesis [28].

## 5 Conclusions

In summary, this experiment successfully established an iBRB cell co-culture model *in vitro* with permeability changes of the iBRB observed by indirect measurement of the TEER, and the model was treated with AGEs to simulate iBRB damage and to detect changes of VEGF and PEDF. Our findings showed that VEGF increased and PEDF decreased, both in a dose- and time-dependent manner, and the balance of VEGF and PEDF was lost in the presence of AGEs in the co-culture model. The increase of permeability shown by the TEER was dependent on the dose and time of AGE intervention, so the imbalance of VEGF and PEDF may be one of the important reasons for the change of permeability of the RMEC layer. Thus, the *in vitro* iBRB model constructed by RMEC and RMGC co-culture can be used for studying the pathogenesis of retinal vascular diseases such as DR for evaluating the impact of candidate drugs, even though the relevant assays *in vivo* remain necessary.
